# Training and evaluating simulation debriefers in low-resource settings: lessons learned from Bihar, India

**DOI:** 10.1186/s12909-019-1906-2

**Published:** 2020-01-08

**Authors:** Julia H. Raney, Melissa M. Medvedev, Susanna R. Cohen, Hilary Spindler, Rakesh Ghosh, Amelia Christmas, Aritra Das, Aboli Gore, Tanmay Mahapatra, Dilys Walker

**Affiliations:** 1 Department of Pediatrics, Stanford University, 725 Welch Rd, MC: 5906, Palo Alto, CA 94304 USA; 2 Department of Pediatrics, University of California San Francisco, 550 16th Street, Box 1224, San Francisco, CA 94158 USA; 3 Maternal, Adolescent, Reproductive, and Child Health Centre, London School of Hygiene and Tropical Medicine, London, UK; 4 College of Nursing, University of Utah, 10 South 2000 East, Salt Lake City, UT 84112 USA; 5 Global Health Sciences, University of California San Francisco, 550 16th St, San Francisco, CA 94158 USA; 6 PRONTO International, State RMNCH+A Unit, C-16 Krishi Nagar, A.G. Colony, Patna, Bihar 80002 India; 7 Care India Solutions for Sustainable Development, 14 Patliputra Colony, Patna, Bihar 800013 India; 8 Department of Obstetrics and Gynecology and Reproductive Services, University of California San Francisco, 1001 Potrero Ave, San Francisco, CA 94110 USA

**Keywords:** Simulation, Debrief., Low-resource., Barriers., Enablers., India.

## Abstract

**Background:**

To develop effective and sustainable simulation training programs in low-resource settings, it is critical that facilitators are thoroughly trained in debriefing, a critical component of simulation learning. However, large knowledge gaps exist regarding the best way to train and evaluate debrief facilitators in low-resource settings.

**Methods:**

Using a mixed methods approach, this study explored the feasibility of evaluating the debriefing skills of nurse mentors in Bihar, India. Videos of obstetric and neonatal post-simulation debriefs were assessed using two known tools: the Center for Advanced Pediatric and Perinatal Education (CAPE) tool and Debriefing Assessment for Simulation in Healthcare (DASH). Video data was used to evaluate interrater reliability and changes in debriefing performance over time. Additionally, twenty semi-structured interviews with nurse mentors explored perceived barriers and enablers of debriefing in Bihar.

**Results:**

A total of 73 debriefing videos, averaging 18 min each, were analyzed by two raters. The CAPE tool demonstrated higher interrater reliability than the DASH; 13 of 16 CAPE indicators and two of six DASH indicators were judged reliable (ICC > 0.6 or kappa > 0.40). All indicators remained stable or improved over time. The number of ‘instructors questions,’ the amount of ‘trainee responses,’ and the ability to ‘organize the debrief’ improved significantly over time (*p* < 0.01, p < 0.01, *p* = 0.04). Barriers included fear of making mistakes, time constraints, and technical challenges. Enablers included creating a safe learning environment, using contextually appropriate debriefing strategies, and team building. Overall, nurse mentors believed that debriefing was a vital aspect of simulation-based training.

**Conclusion:**

Simulation debriefing and evaluation was feasible among nurse mentors in Bihar. Results demonstrated that the CAPE demonstrated higher interrater reliability than the DASH and that nurse mentors were able to maintain or improve their debriefing skills overtime. Further, debriefing was considered to be critical to the success of the simulation training. However, fear of making mistakes and logistical challenges must be addressed to maximize learning. Teamwork, adaptability, and building a safe learning environment enhanced the quality enhanced the quality of simulation-based training, which could ultimately help to improve maternal and neonatal health outcomes in Bihar.

## Background

Simulation-based training for health providers is becoming widely recognized as a tool for improving facility-based care of mothers and neonates globally [1]. Post-simulation debriefs, where learners identify clinical weaknesses, discuss team functioning, expand their knowledge base, and subsequently apply lessons learned to real cases, is the cornerstone of the learning process [2]. The World Health Organization (WHO) recommends that simulations be added to quality improvement trainings to help address skill gaps [1]. Several programs, including PRONTO (Programa de Rescate Obstétrico y Neonatal: Tratamiento Óptimo y Oportuno) International [3], Jhepiego [4], and Helping Babies Breathe (HBB) [5] have implemented simulation-based maternal and neonatal training programs in low- and middle-income countries (LMIC), including Mexico [6], Guatemala [7], Tanzania [8], and India [9], These programs have demonstrated improvements in clinical skills and in 24-h neonatal survival [10]; however, several critical implementation questions remain. In low-resource settings, how do you support and sustain debriefing competency, the most challenging skill of simulation facilitation? How is this best done at scale?

Debrief facilitation of simulations is difficult to learn, and achieving fluency and expertise requires time and experience [2]. For simulation to have its optimal effects, an experienced facilitator guides reflective learning, creates a safe learning environment, and encourages self-reflection [11, 12]. Several debrief evaluation tools have been designed, validated, and implemented in high-resource settings for simulations [13–16]. These tools provide valuable feedback, which is critical for facilitator development and for enhancing of the learning experience of future simulation participants.

Despite the known importance of effective debriefing and the growing demand for simulation-based training globally, the optimal way to train and evaluate simulation facilitators in low-resource settings is unknown [17]. Recent research has highlighted the complex role that culture plays on debrief facilitation. In low-resource settings, two important challenges exist. First, facilitators generally have limited to no previous experience with simulation-based training and rely heavily on unilateral, didactic approaches. A multi-country study demonstrated that debrief facilitators from high-power difference (i.e., hierarchical) cultures were less likely to ask open-ended questions and more likely to talk rather than facilitate discussion [18]. Second, health facilities in many LMIC settings lack a culture of non-punitive feedback, a key component of successful debriefing. In the Rwanda Emergency Triage, Assessment and Treatment plus admission care (ETAT+) trial, authors reported that reviewing mortality data with trainees was difficult because this practice made trainees feel shameful [19]. A HBB training program in Guatemala found that debriefing was a new concept for participants and suggested increased training time focused on debriefing methods and feedback for future participants [20]. In Bihar, a predominately rural Indian state with very low socioeconomic status [21] and a largely didactic model of education [22], such challenges are likely more pervasive.

Given the rapid growth of simulation-based training in low-resource settings, it is critical to have tools to accurately evaluate the debriefing abilities of facilitators. This knowledge will allow simulation programs to provide feedback to help facilitators improve their skills and maximize trainee learning. The Debriefing Assessment for Simulation in Healthcare (DASH) tool, developed at the Harvard Simulation Center, is the most widely used debrief evaluation tool and has been extensively validated in high-resource settings [13]. The DASH tool evaluates instructors on key behaviors that facilitate learning and change using six behavioral components [23]. The Center for Advanced Pediatric and Perinatal Education (CAPE) Debriefing Evaluation Tool was developed at Stanford University [14]. Compared to the DASH tool, the CAPE tool uses more objective criteria, which we hypothesize may be more accessible to less experienced debrief facilitators in LMICs. The aim of this study was to explore debrief training and evaluation in Bihar by i) evaluating the interrater reliability of the CAPE and DASH tools using video-recorded debriefing sessions conducted during simulation trainings, ii) assessing changes in nurse mentors’ debriefing skills over time, and iii) exploring barriers and enablers of simulation debriefing among nurse mentors.

## Methods

### Study setting

Bihar has a population of over 100 million, with 89% living in rural areas [24]. In 2012, the maternal mortality rate (MMR) was 208 per 100,000 live births in Bihar and the neonatal mortality rate (death within the first days) was 34 per 1000 live births [25]. In Bihar, each block primary health center (PHC) serves an average population of ~ 190,000. One nurse is frequently responsible for all obstetric and neonatal delivery care at a given PHC [26].

### Study design

This was a mixed methods study, including quantitative and qualitative data.

### Program overview

The Mobile Nurse Mentoring Program, AMANAT (meaning “something given in trust”), was a large-scale obstetric and neonatal nurse mentoring program led by CARE India in collaboration with the Government of Bihar. The AMANAT program was implemented at 320 PHCs across Bihar between 2015 and 2017 over four 8-month rounds. Rounds 1–3 were included in this analysis as Round 4 was ongoing. During each round, nurse mentors rotated in pairs between four sites, spending one week per month at each site for a total of six to eight weeks at each PHC. Each PHC had six to eight nurse mentees. Starting in week 3 on their third visit, nurse mentors facilitated a minimum of three simulations per week focused on key maternal and neonatal scenarios. Each simulation was followed by a debrief, which was recorded using a handheld camera.

### Study population

A total of 120 nurse mentors participated in AMANAT rounds 1 through 3, which were conducted from March 2015 to June 2016. Nurses were selected by CARE India and the Government of Bihar to work as on-site mentors and simulation facilitators. The details of this program are described elsewhere [27]. Nurse mentees were nurses working in PHCs in Bihar, who were required to have an Auxiliary Nurse Midwife (ANM) or General Nursing and Midwifery (GNM) qualification. ANM and GNM qualifications require a secondary education with an additional two or three and a half years of nursing training, respectively.

### Simulation facilitation training

Nurse mentor training was implemented using the train-the-trainer approach. Nurse mentors underwent four weeks of in-depth training with CARE India. One week was entirely devoted to Basic Emergency Obstetric and Neonatal Care (BEmONC) simulation training with PRONTO International. This training included simulation facilitation, teamwork, communication, and debriefing skills. One half-day focused exclusively on the theory of debriefing and 1.5 days allowed for the practice of debriefing skills. Nurse mentors were taught to facilitate debriefs using the diamond debriefing method, a structure that includes three phases: description, analysis, and application [28]. This approach encourages participants to reflect on their behavior, review practice guidelines, focus on teamwork and communication (based on TeamSTEPPS™) [11], and consider how to apply knowledge and skills to real-life clinical practice. Throughout the training, the key concepts from the CAPE and DASH, particularly the importance of facilitating discussion rather than lecturing, was emphasized. Nurse mentors were provided a menu of 31 SimPacks™ (simulation scenario and debriefing guide) from which they could choose. Due to time constraints, mentors did not receive individualized feedback from videos until after the round had been completed; however, four months following the initial training, nurse mentors completed an additional four-day Advanced Simulation Facilitator training with PRONTO, which focused on simulation facilitation and debriefing skills [29].

### Part 1. Evaluating inter-rater reliability of the DASH and CAPE tools

#### Debrief monitoring

To evaluate debriefing quality, the research team randomly selected one debriefing video per mentor pair during three time points: early (months 3–4), mid (month 5), and late (months 6–7). The target sample size was 85, based on the suggestion of Bujang and Baharum that 85 items are required when the null hypothesis can be assumed to not equal zero and there are two observations per subject [30]. This sample size provides adequate power for estimating Cohen’s Kappa with 2 raters per item [31]. This study included debriefs of normal spontaneous vaginal delivery (NSVD), postpartum hemorrhage (PPH) and neonatal resuscitation (NR) simulation scenarios. Debrief videos were analyzed using the DASH and CAPE tools. These two tools were modified by a group of low-resource simulation experts at University of California San Francisco (UCSF), PRONTO, and University of Utah, with the input of clinical providers in Bihar. We made two modifications to the DASH tool. First, the 1–7 Likert scale was reduced to a 1–5 scale because evidence from the literature suggests higher data validity with 1–5 scales if respondents have variable levels of education [32]. Second, the first element of the DASH tool, ‘establishes and engaging learning environment,’ was skipped because the pre-debrief was not filmed and therefore could not be evaluated [23]. The DASH forms were then inputted into Qualtrics™ surveys for electronic data collection. Several modifications to the CAPE tool were also made. Due to logistical challenges, the following indicators were removed: ‘time between end of scenario and start of debriefing;’ ‘time when audio first rolls during debriefing;’ ‘length of debriefing to length of scenario ratio;’ and ‘percentage of scenario covered during debrief.’ The indicator, ‘percent of learning objectives covered during debriefing,’ was adjusted to reflect the ‘total number of cognitive, technical, and behavioral objectives covered’ in order to simplify coding. Finally, a code window of the modified CAPE tool (Appendix 1) was developed using Studiocode™^.^ video coding software.

Two nurses (henceforth called video analysts), both based in Bihar, not involved in program implementation, and fluent in Hindi (the local language), were trained in debrief video analysis. This training consisted of a two-hour lesson on debrief theory, a detailed review of the modified DASH and CAPE tools, and coding in Studiocode™^.^. During rater training, the video analysts and one Hindi-speaking simulation expert triple-coded 10 debrief videos. Each watched the videos twice, first completing the modified DASH form and then the CAPE code window. With the guidance of a PRONTO expert trained in DASH evaluation, any resulting discrepancies were discussed and resolved. This process was repeated until the PRONTO expert trained in DASH-evaluation determined that both raters demonstrated proficiency with the DASH and CAPE constructs. Additionally, the video analysts participated in biweekly calls throughout the course of the project to review progress and discuss coding-related questions.

#### Statistical analysis

All videos selected were of sufficient quality to analyze. Any missing individual responses were excluded from analysis, with the exception of certain CAPE variables that asked about the presence of a certain component (i.e., ‘Is the analysis phase present?,’ ‘Number of times the video was paused?’). In these cases, a missing response was replaced with a zero. All videos were double-coded by the two video analysts. To mitigate rater bias, the video files presented to the video analysts were given in batches. Intra-class correlation coefficients (ICC)^1^ with 95% confidence intervals (CI) were calculated for continuous CAPE variables and DASH elements. Variables lacking normal distribution were log-transformed prior to ICC calculation. ICCs < 0.40, 0.40–0.59, 0.60–0.74, and ≥ 0.75 were considered poor, fair, good, and excellent, respectively [33]. Reliability for binary variables was assessed using Cohen’s kappa with 95% CIs, with levels of agreement < 0.40, 0.40–0.70, and > 0.75 considered low, fair to good, and excellent, respectively [34]. To assess the internal consistency of the elements of the DASH scale, Cronbach’s α was calculated for both raters using all double-coded videos. Cronbach’s α was not calculated for the CAPE tool, as it contains continuous variables [35].

### Part 2. Assessing changes in nurse mentors’ debriefing skills over time

Changes in nurse mentors’ debriefing skills were evaluated over each 8-month round, using unpaired debriefing videos. We hypothesized that mentors’ skills would improve over time secondary to increased practice, strengthened relationships with their learners, and the simulation refresher training conducted after month four. Only indicators that were found to have fair to excellent interrater reliability were included in the analysis to assess change over time, a decision that was made a priori to maximize accuracy. Depending on the timing of the debriefs, videos were categorized into three time-points: early (months 3–4), mid (month 5), and late (months 6–7). Trends over time for all continuous and categorical variables were assessed using linear and logistic regression, respectively, adjusted for rater. Because there was significant variation between which of the two mentor pairs led the debrief at each timepoint, a paired analysis was not possible. In more conservative models, we used generalized estimating equations (GEE) to account for correlations between double coding by raters per video. The GEE and non-GEE models yielded similar results. For simplicity of interpretation, only linear and logistic models are reported, except when differences were observed. Regression assumptions, including normality, homoscedasticity, outlier and influential analysis, were examined to detect any potential violations. All analyses were conducted in R Core Team version 0.99.903 (R Foundation for Statistical Computing, Vienna, Austria) [36].

### Part 3. Exploring barriers and enablers of debriefing among nurse mentors

We explored barriers and enablers of simulation debriefing through semi-structured interviews with current AMANAT nurse mentors. Interviews took place between June and August 2016. The interview guide was designed in English, translated into Hindi, and then translated back to English to ensure accuracy. Two pilot interviews were completed to refine the interview guide. The pilot interviews were excluded from the final analysis. Interviews were conducted by two female interviewers, who had received training on study objectives and qualitative methodology. One interviewer was fluent in Hindi. Interviews were conducted in the language preferred by participants. Interviews were held in private rooms at PHCs. Interview duration ranged from 40 to 60 min.

#### Thematic analysis

Interviews were transcribed and, where necessary, translated to English by a bilingual Indian simulation specialist. To ensure transcription and translation accuracy, two independent staff double-checked all transcriptions and translations. Interview data were analyzed using the thematic content approach, which included four steps: data familiarization; identifying codes and themes; developing a coding scheme and applying it to the data; and organizing codes and themes [37, 38]. Two interviews were double-coded by the second author and another co-author. Any discrepancies were discussed and resolved to develop the final coding framework. The second author coded all remaining transcripts.

### Ethics

Written informed consent was obtained from all participants. The study was approved by the UCSF Committee on Human Research (Approval# 14–15,446) and the Indian Institute of Health Management Research Institutional Review Board.

## Results

### Part 1. Evaluating the interrater reliability of the DASH and CAPE tools

Across three mentoring phases from March 2015 to June 2016, 4066 simulation debrief videos were collected. A total of 73 debrief videos were included in the analysis (Table 1).

**Table 1:** Characteristics of debrief videos included in the analysis

Round	Total Debrief Videos	Coded Debrief Videos by Study Timepoints		
		Early Months 3–4	Mid: Month 5	Late: Months 6–7
1	1412	14	8	11
2	1648	14	0	0
3	1006	5	10	11
ALL	4066	33	18	22

Overall, the CAPE tool had high interrater reliability than the DASH tool. Eight CAPE indicators had excellent interrater reliability (50%), while only 3 of 16 (19%) demonstrated poor reliability (Table 2). In comparison, 3 of 5 DASH elements had poor reliability and none had excellent reliability.

**Table 2:** Interrater reliability of CAPE and DASH variables in Bihar, India, 2015–2017 (*N* = 73 simulation debrief videos)

Indicator	N	Reliability	Level
CAPE Tool			
Communication			
Instructor questions	73	0.52 (0.24–0.69) ^§^	Fair
Instructor statements	73	0.14 (−0.36–0.46) ^§^	Poor
Trainee responses	73	0.96 (0.93–0.97) ^§^	Excellent
Instructor questions to instructor statements ratio	73	0.19 (−0.29–0.49) ^#^	Poor
Trainee responses to instructor questions + statements ratio	73	0.77 (0.63–0.85) ^§^	Excellent
Objectives			
Behavioral objectives mentioned	73	0.67 (0.48–0.79) ^§^	Good
Cognitive objectives mentioned	73	0.34 (0.04–0.59) ^§^	Poor
Technical objectives mentioned	73	0.35 (0.07–0.64) ^#^	Fair
Structure			
Length of debrief video	71 ^α^	1.0 (0.99–1.0) §	Excellent
All three debrief structural phases present	73	0.72 (0.56–0.25) ^‡^	Good
Length of description phase	73	0.78 (0.65–0.86) ^#^	Excellent
Length of analysis phase	73	0.92 (0.87–0.95) ^§^	Excellent
Length of application phase	73	0.67 (0.48–0.79) ^#^	Good
Video Use			
Incorporation of simulation video	73	1.0 (1.0–1.0) ^‡^	Excellent
Length of tape segments played	73	0.98 (0.97–0.99) ^§^	Excellent
Number of times tape paused during	73	0.99 (0.98–0.99) ^#^	Excellent
DASH Tool			
Elements			
1: Maintain engaged learning environment	71 ^α^	0.26 (−0.18–0.54) ^§^	Poor
2: Organize the debrief	73	0.59 (0.34–0.74) ^§^	Fair
3: Facilitate discussion	72 ^α^	0.64 (0.43–0.77) ^§^	Good
4: Identify growth opportunities	72 ^α^	0.38(0.02–0.61) ^§^	Poor
5: Create success plan	72 ^α^	0.22 (−0.25–0.51) ^§^	Poor
Mean DASH score	68 ^α^	0.37 (0.02–0.61) ^§^	Poor

§ ICC calculated for continuous variables (95% CI).

# ICC calculated from normalized data (95% CI).

‡ Cohen’s kappa calculated for binary variables (95% CI).

α Some forms had missing data.

One of the most important CAPE indicators, ‘Instructor questions to instructor statements ratio,’ was not reliably coded. However, a composite indicator, ‘instructor questions and statements,’ demonstrated high reliability (data not shown).

Only two DASH indicators, ‘organize the debrief’ and ‘facilitate the debrief,’ demonstrated fair and good reliability, respectively; four of six (66%) demonstrated poor reliability (Table 2). Cronbach’s α of the DASH tool was 0.96 and 0.95 for raters 1 and 2, respectively.

### Part 2. Assessing changes in nurse mentors’ debriefing skills over time

Following training, nurse mentors’ performance increased for several reliable CAPE and DASH indicators that are key to the essence of the debrief quality (Table 3). The average number of ‘instructor questions’ increased from 34 to 49 per debrief (*p* < 0.01). The number of ‘trainee responses’ increased from 50 to 64 per debrief (p < 0.01). The DASH indicator, ‘organize the debrief,’ increased from 3.3 to 3.5 (*p* = 0.04) on a 5-point Likert scale.

**Table 3:** Changes in nurse mentors’ debriefing skills over time in Bihar, India, 2015–2017 (*N* = 73 simulation debrief videos)

		Early: months 3–4 *n* = 33	Mid: month 5 *n* = 18	Late: months 6–7 *n* = 22	
Indicator	N	Mean (SD*)			*p*-value
CAPE Tool					
Communication					
Instructor questions	73	34 (22)	48 (26)	49 (27)	< 0.01 ^§^
Trainee response	73	50 (24)	69 (25)	64 (27)	< 0.01 ^§^
Trainee responses to instructor questions + statements ratio	73	0.75 (0.15)	0.79 (0.16)	0.78 (0.15)	0.20 ^§^
Structure					
Video Length	71	17.6 (8.1)	20.3 (8.7)	17.8 (8.1)	0.74 ^§^
Length of description phase (min)	73	1.0 (0.8)	1.0 (0.5)	1.1 (0.8)	0.83 ^§^
Length of analysis phase (min)	73	13.7 (8.5)	17.4 (8.9)	15.6 (7.4)	0.18 ^§ ‡^
Length of application phase (min)	73	1.1 (0.8)	1.4 (1.1)	0.8 (0.9)	0.22 ^§^
All three phases present (%)	73	71 (46)	86 (35)	61 (49)	0.37 ^#^
Objectives					
Behavioral objectives mentioned	73	5.6 (4.0)	7.0 (4.0)	6.3 (4.9)	0.33 ^§^
Technical objectives mentioned	73	3.3 (3.7)	3.4 (4.0)	3.1 (2.9)	0.78 ^§^
Video use					
Use of video playback (%)	73	85 (36)	89 (32)	73 (45)	0.14 ^#^
Number of times tape paused during	73	6.7 (6.7)	8.8 (4.8)	5.6 (5.8)	0.48 ^§^
Length of tape segments played (min)	73	4.5 (3.4)	4.3 (2.5)	3.3 (2.9)	0.06 ^§^
DASH					
Elements					
2: Organize the debrief	72	3.3 (1.0)	3.8 (0.7)	3.5 (0.9)	0.04 ^§ α^
3: Facilitate discussion	72	3.4 (0.7)	3.8 (0.7)	3.6 (0.8)	0.14 ^§ #^

§ Linear regression adjusting for rater.

# Logistic regression adjusting for rater.

* Standard Deviation (SD)

‡ This value was not significant at the 0.05 in the GEE model.

The majority of indicators did not change over time. For example, ‘trainee response to instructor questions and statements ratio’ changed from 0.75 to 0.78. The ‘number of times the videotape was paused’ during debriefing, ‘debrief length’, and ‘number of behavioral and technical objectives’ mentioned all remained constant (*p* > 0.05).

No indicators decreased significantly over time, though some trended downwards. The ‘length of tape segment played’ decreased from 4.5 to 3.3 min, and ‘use of video playback’ decreased from 85 to 73%. Additionally, 71 and 61% of debriefs had ‘all three phases present’ during month 1 and months 6–7, respectively. The most commonly omitted phase of the debrief was ‘application’ (data not shown).

### Part 3. Exploring barriers and enablers of debriefing among nurse mentors

A total of 20 nurse mentors, with a median age of 24 years, were interviewed. On average, they had 14 months of mentoring experience. Only three had previous teaching experience and none had prior simulation debriefing experience. Participants were from states across India, including Delhi [7], West Bengal [4], Kerala [3], Bihar [2], Maharashtra [2], Uttar Pradesh, and Orissa [1].

### Barriers

#### Uncomfortable discussing mistakes

Many participants described that mentees disliked having their mistakes identified, especially when these mistakes were captured on video. Mentees, particularly older nurses, worried that such videos would be used to publicly display mistakes to peers. While mentors acknowledged that the videos sometimes made mentees nervous, they found them helpful in providing feedback.

*"It should be good... continuing with the video, because the person... if they are doing mistake, they can observe, 'Oh yeah, they are doing.' According to me, the video should be there."* (Mentor, age 22)

To mitigate anxiety, mentors tried to reassure mentees that the videos were only learning tools.

#### Time management

Participants commonly struggled with time management during debriefing. Several mentioned that it was difficult to keep all of the mentees engaged when debriefs were longer than 20 to 30 min. Challenges included mentees exhibiting disinterest, talking simultaneously, and arguing about clinical management. Further, because mentees were frequently scheduled to work on training days, they sometimes had to leave debrief sessions to care for patients.

#### Technical challenges

Several mentors described technical barriers related to video-recording. When the video, camera or laptop was not working, mentors often used mobile phones to record videos.

### Enablers

#### Create a positive learning environment

Numerous mentors highlighted the importance of creating a safe learning environment for mentees. To do this, mentors would begin debriefs by discussing what went well. Mentors framed mistakes in a constructive way and encouraged mentees to self-identify how they could improve in the future. Additionally, mentors emphasized the importance of using supportive language.

*"First of all, we used to take the positive points what all they have done. Actually, I didn't used to take the negative points... I used to ask them what [they] could have done at this place."* (Mentor, age 24)

*“We say... 'Sister, you tell us what got missed and what should you have added,' so then she herself will tell her mistakes. Through this, what happens is that we are on the safe side. In the beginning, they used to feel very guilty... 'Sister, I made a mistake... the mistake has been recorded in the video.' Then we say, 'Sister, let mistakes happen, only then we can learn from them, but don’t repeat them.'"* (Mentor, age 24)

*“Don't call out mistakes. [Instead] say 'missed out.'”* (Mentor, age 24)

#### Contextually appropriate debriefing

Several participants discussed the importance of being efficient and organized during debriefs. Mentors tried to keep debriefs as short as possible, while still covering the key points. At times, this required group management skills; for example, “If you sit, it will be done” (Mentor, age 26). When PHCs were really busy, mentors utilized flash debriefs. These pre-written debrief scripts consisted of 3 questions (what when well, what could have gone better, what will you do next time you encounter a similar clinical scenario) and rapidly covered the most important messages for a given simulation scenario.

#### Team building

Mentors also discussed strategies to increase the participation of mentees and other PHC providers in debriefing sessions. The majority of mentors recommended including doctors in simulations and debriefs. Additionally, several suggested beginning the debriefs with mentees summarizing the preceding simulation scenario.

### Overall perception

Nearly all mentors had a positive perception of debriefs, describing them as a critical element of simulation training.

*"If we do not debrief, there is no point of simulation."* (Mentor, age 22)

A majority of mentors believed that debriefs helped clarify clinical weaknesses, so that mistakes that occurred during simulations would not happen while taking care of patients.

*"I think debriefing is like the backbone of simulation... because with debriefing, they used to understand everything they did not understand well with the simulation… if they used to think that. ‘I have done this well,’ then in debriefing they used to realize that, ‘No, I could not do it.’ If they feel that I have done any mistake then... it used to get clear in the debrief."* (Mentor, age 22)

Mentors also felt that debriefing was valuable for improving provider communication, discussing doctor-nurse and nurse-nurse hierarchy, and identifying other health system-related challenges such as human resource shortages and long distances between the delivery room and the pharmacy where necessary medications are kept.

## Discussion

To develop effective and sustainable simulation training programs in low-resource settings, it is critical that facilitators are thoroughly trained in debriefing. Through this unique approach using video analysis, we were able to remotely monitor and evaluate simulation debriefing in Bihar. Results suggest that the CAPE tool more reliably assessed debriefs, compared to the DASH tool. Thirteen of the 16 CAPE indicators had fair to excellent reliability (81%). This may partially be related to the fact that the DASH evaluates skills at the composite level, whereas the CAPE, which is scored at the individual item level, does not. Notably, this finding suggests that the CAPE tool’s objectivity may be especially helpful in settings where evaluators have less experience evaluating debriefers. One key indicator, ‘ratio of instructor questions to statements,’ had low reliability. However, a composite indicator of the sum of ‘instructor questions and statements’ was highly reliable, suggesting that the two video analysts were systematically categorizing questions and statements differently. For example, one video analyst was coding rhetorical questions as a statement, while the other was not. The DASH tool demonstrated high internal consistency with a Cronbach α of > 0.95, which is higher than the original high-resource validation study that found a Cronbach α of 0.89 [13]. This could suggest that the analysts scored each DASH question similarly and did not understand the different elements of the tool [39]. All indicators with high interrater reliability increased or were maintained over the 8-month mentoring period. This suggests that, as mentors improved their facilitation skills, mentees were empowered to develop the confidence required to discuss performance in simulations with peers. However, a significant improvement in mentor debriefing skills over time was not identified. This highlights the need for more timely and frequent debriefing feedback as well as revision of the debrief evaluation tools to better reflect the context in which mentors are working. Nevertheless, in a culture that largely utilizes a traditional didactic model of teaching [22], these findings represent meaningful progress.

Mentors identified fear of making mistakes, time-constraints, and technical challenges as key challenges to successful debriefing. Previous studies have similarly identified lack of protected time for professional development [40] and lack of feedback culture [17, 19] as significant barriers to provider training in LMIC settings. A Rwandan study found that providers who attended training outside of their usual workplace, where they were guaranteed to be free of clinical duties, had two-fold increased odds of passing practical skills assessments compared to providers who completed training in their workplace [41]. A multi-country study found that Asian simulation participants were often uncomfortable correcting other participants, especially those in authority positions, for fear of causing shame or appearing oppositional [42].

Interviews revealed several approaches to address identified barriers to enable success in this resource-constrained context. While mentees initially felt shameful about mistakes, mentors increased participation by constructively framing mistakes as learning points. This thoughtful attention to language allowed mentees to feel comfortable discussing mistakes, while still maintaining a respectful learning environment.

Findings suggest that contextually appropriate flash debriefs, which may be easily adapted to reflect trainee needs, could help overcome the important barrier of time-constraints, though future studies are required to explore whether these are equally effective from a learning perspective. This is consistent with previous studies that have recommended adaptation of debriefs to fit the environment and skill level of trainees [12]. Acceptability of this flexible approach to debriefing is critical, as government-run PHCs in India often face severe human resource shortages and, as a result, clinical duties are routinely prioritized over training [24, 43]. Additional recommendations related to increasing group participation and including doctors in both simulations and debriefs. A previous study in Bihar similarly suggested that inclusion of doctors in simulation training leads to improved communication and reduced hierarchy in PHCs [43]. This is consistent with previous studies in Rwanda and Kenya that highlighted the importance of teamwork [44] and leadership buy-in, respectively [45].

This study has several limitations. First, the video analysts did not participate in the official DASH training due to time and financial constraints. This may have been an important contributor to the low interrater reliability reported in this study. The number of debrief videos analyzed from round 2 was relatively small as a result of missing data from a third video analyst, who left after a brief period of employment; this may have resulted in an underestimation of interrater reliability or failure to detect changes in debriefing performance. Video coding was both time- and resource-intensive. Finally, interviews were conducted by members of the study team, which may have introduced social desirability bias. All mentors were informed in advance that data resulting from interviews was confidential in nature and would not be used for purposes other than research and programmatic improvement.

## Conclusion

This study has demonstrated the feasibility of evaluating simulation debriefing in Bihar, India. Multiple CAPE indicators reliably assessed debriefing performance, showing that nurse mentors maintained or improved their facilitation skills over time. Barriers included fear of mistakes and time constraints. Enablers included having a safe learning environment, a flexible approach to debriefing, and leadership buy-in. An in-depth understanding of the barriers and enablers of debriefing is essential to improve the quality of simulation training programs in LMICs. Establishing the feasibility of debriefing and debrief evaluation is a meaningful step toward the development of successful simulation training programs and ultimately improving BEmONC skills among providers in Bihar and related low-resource settings.

## Supplementary information

**Additional file 1: Table S1** Interrater reliability of additional CAPE variables in Bihar, India, 2015–2017 (*N* = 73 simulation debrief videos).

## Data Availability

Analyses are ongoing so the data is not yet publically available.

## Footnotes

1 Model 1 with k raters
