# Supplementary material for: Training and evaluating simulation debriefers in low-resource settings: lessons learned from Bihar, India
Source: BMC Med Educ. 2020 Jan 8;20:9. doi: 10.1186/s12909-019-1906-2 (PMC6951007; doi:10.1186/s12909-019-1906-2)
Supplement: Supplementary file 1 — Additional file 1: Table S1 Interrater reliability of additional CAPE variables in Bihar, India, 2015–2017 (N = 73 simulation debrief videos). [file 12909_2019_1906_MOESM1_ESM.docx]

| Indicator |  | Reliability | Level |
| --- | --- | --- | --- |
| CAPE: communication | Mean |  |  |
| Instructor questions + statements | 78 | 0.93 (0.90 – 0.96) ^§^ | Excellent |
|  |  |  |  |
| CAPE: structure | Percent |  |  |
| Descriptive phase included | 97% | 0.79 (0.4 – 1.0) ^‡^ | Excellent |
| Analysis phase included | 97% | -0.05 (-0.01 0.01) ^‡^ | Poor |
| Application phase included | 75% | 0.53 (0.31-0.75) ^‡^ | Excellent |

§ ICC calculated for continuous variables (95% CI)

‡ Cohen’s kappa calculated for binary variables (95% CI)
